# Pain management strategies and their associations with quality of life and pain interference in women with chronic pelvic pain: A cross-sectional survey

**DOI:** 10.1177/17455057261485419

**Published:** 2026-10-07

**Authors:** Darya Ghodosi, Jasmine Heath Hearn

**Affiliations:** 1 5289 School of Psychology, Manchester Metropolitan University, Manchester, United Kingdom

**Keywords:** chronic pelvic pain, women, pain management, quality of life, pain interference, endometriosis

## Abstract

**Background:**

Chronic pelvic pain (CPP) affects an estimated 6-27% of women and is associated with substantial impairment in quality of life (QoL). Common conditions include endometriosis, vulvodynia, and interstitial cystitis. Management typically involves multiple biomedical and psychological strategies, often used in combination, yet their independent associations with QoL and pain interference remain unclear.

**Objectives:**

To examine associations between six pain management strategies (surgery, hormonal medication, non-hormonal medications/therapies, analgesia, alternative therapies, and psychological therapy) and World Health Organization Quality of Life (WHOQOL-BREF) domains, and pain interference in women with CPP.

**Design:**

Cross-sectional survey study.

**Methods:**

Women with CPP (N = 147) completed an online questionnaire. Outcomes included WHOQOL-BREF Physical, Psychological, Social, and Environment domains, global QoL, and pain interference. Multiple linear regression models examined associations with QoL domains and pain interference, adjusting for simultaneous strategy use. Ordinal logistic regression examined global quality of life.

**Results:**

Surgery was associated with lower Physical QoL (p = .007). Analgesia was associated with poorer Physical (p = .033), Psychological (p = .022), and Environmental quality of life (p = .041). Psychological therapy was associated with higher Psychological (p = .037) and Environment QoL (p = .023). Use of non-hormonal medications/therapies was associated with greater pain interference (p = .016). No clear associations were observed for Social QoL or global QoL.

**Conclusion:**

Overall patterns are consistent with a biopsychosocial understanding of CPP. Surgery and analgesia were associated with lower QoL in selected domains, which may reflect greater symptom severity, treatment complexity, or confounding by indication. Psychological therapy was associated with higher psychological and environmental QoL while non-hormonal medications/therapies suggested greater pain interference. Findings suggest earlier integration of psychological care, regular review of analgesic use, and routine monitoring of quality of life and pain interference within multidisciplinary CPP pathways. Causal inferences are limited by the cross-sectional design.

## Introduction

Chronic pelvic pain (CPP) is a complex, debilitating pain in the pelvic region that is persistent, non-cyclical or intermittent, lasts three months or longer, and is severe enough to impair work, daily functioning, or social life.^1^ Common causes of pelvic pain include endometriosis, pelvic inflammatory disease, vulvodynia, and interstitial cystitis, but CPP can also present in the absence of an identifiable cause.^2,3^ CPP affects physical and emotional health and is linked with disability and reduced quality of life (QoL).^4^ Estimates of CPP prevalence among women vary from approximately 6% to 27%, reflecting methodological differences across definitions, populations and measurement approaches.^5^ Symptom overlap with disorders such as irritable bowel syndrome often delays diagnosis and treatment, prolongs suffering, and increases healthcare-associated costs.^6^ As a result, in the UK, the average time to diagnosis remains long and variable, indicating fragmented pathways to care and compounding physical, psychological and social impacts.^7^

National reviews identify unmet support needs for CPP. The 2024 National Confidential Enquiry into Patient Outcome and Death reported extensive diagnostic journeys and low rates of multidisciplinary discussion, which suggest current services do not meet the needs of people with CPP.^8^ Endometriosis UK reported that the average diagnosis time has risen to nearly nine years, with many patients seeing a GP multiple times before referral for diagnostic testing or further treatment.^9^ While psychological approaches such as cognitive behavioural therapy (CBT) and acceptance and commitment therapy (ACT) have been evaluated in CPP and wider chronic pain populations, they are not consistently embedded within routine gynaecology and pelvic-pain pathways. For example, NICE guidance for endometriosis emphasises surgical and pharmacological care but provides limited direction on psychological therapies for pain management,^10,11^ despite evidence that such interventions are efficacious in improving pain-related outcomes such as disability in chronic pain in other contexts (e.g. chronic low back pain).^12^ Given the lengthy diagnosis delays and inconsistent care pathways, understanding pain management strategies used by people with CPP and their impact on pain and quality of life (QoL) are essential.

In this study, pain-management strategies were considered as broad categories reflecting common approaches reported by patients: surgical treatment, hormonal therapies, non-hormonal medications or therapies, analgesia, alternative therapies and psychological therapies.

A range of pain management strategies for CPP are used in practice, often in combination, with inconsistent evidence across treatment types. Surgery, particularly excision or ablation of endometriotic lesions, is commonly offered. Evidence suggests excision outperforms ablation for pain reduction, although recurrence remains common, and outcomes vary with surgical expertise and follow-up care.^13–15^ Postoperative hormonal suppression can reduce recurrence, but patient adherence and tolerability vary.^16^ Studies using validated QoL instruments report average improvements after excision that appear larger in patients with less central sensitisation.^17^ Overall, surgical care can help some patients but often needs to be combined with rehabilitation and psychosocial support.

Hormonal therapies, including combined oral contraceptives and progestins, are guideline-recommended for endometriosis and some gynaecological contributors to CPP. They reduce cyclical pain for many patients but their effects on broader QoL depend on side effects, fertility goals, and coexisting mechanisms such as pelvic floor myalgia or nociplastic pain 10. Discontinuation rates are high and adverse effects such as mood change, bone density loss, and weight gain influence adherence.^18^

Non-hormonal medications/therapies and analgesia are widely used. Non-steroidal anti-inflammatory drugs may be used, yet sustained efficacy in CPP is uncertain and risks accumulate with long-term use.^19,20^ Neuropathic agents such as duloxetine and amitriptyline may benefit some, while a large trial found no benefit of gabapentin for CPP without clear pathology.^21^ Analgesics, including opioids, can relieve pain in the short term, yet long-term data often show small or negative effects on function and QoL and increased risks, especially when combined with gabapentinoids.^22–24^ NICE advises against opioids for chronic primary pain and recommends review and deprescribing where appropriate.^11^ Cross-sectional work often finds greater analgesic use in patients with higher pain severity and side-effect burden, which can relate to lower psychological and environmental QoL.^23^

Psychological therapies including CBT and ACT show small to moderate improvements in disability, mood, and quality of life in chronic pain populations, including women with chronic pelvic pain ^25,26^. Trials in endometriosis also report QoL gains including improvements in quality of life and mental health outcomes even when pain persists for some participants.^27^ These therapies may also support reduced catastrophising, increased acceptance, and greater psychological flexibility.^28^ However, referral and access in gynaecology care pathways remains inconsistent.^29^

There is growing evidence to support the use of physiotherapy and mind-body approaches in CPP management. Pelvic floor physiotherapy and multimodal programmes that combine manual therapy, exercise, and education achieve clinically meaningful improvements in pain and sexual function in recent meta-analyses.^30,31^ Mindfulness and yoga can reduce pain interference and improve coping, although evidence for acupuncture and other complementary therapies remains mixed.^31,32^

Most research isolates single treatments, whereas patients can often engage with multiple pain management strategies, and as such may limit understanding of patient experience and outcomes.^32,33^ A multivariable approach that allows overlapping strategy use can test the unique associations of surgery, hormonal therapy, non-hormonal medications/therapies, analgesia, physiotherapy or complementary approaches, and psychological therapy with quality of life and pain interference. This approach aligns with UK calls for multidisciplinary, non-pharmacological options and can inform service redesign.^10,11^

### Study aims

1. To examine associations between six pain-management strategies (surgery, hormonal therapies, non-hormonal medications/therapies, analgesia, alternative therapies, and psychological therapies) and pain interference in women living with CPP.
2. To examine associations between these strategies and QoL domains.

## Methods

### Design

This cross-sectional quantitative survey examined associations between pain-management strategies, pain interference, and QoL in women living with CPP. Data was collected using Qualtrics.^34^ This cross-sectional study was reported in accordance with the Strengthening the Reporting of Observational Studies in Epidemiology (STROBE) guidelines.^35^

### Participants and recruitment

Purposive and convenience sampling was used. Inclusion criteria were: age 18 years or older, assigned female at birth, CPP for at least three months (with or without formal diagnosis), and self-reported use of at least one pain-management strategy: surgery, hormonal medication or therapies, non-hormonal medications/therapies, analgesia, psychological therapies, or alternative therapies. Participants were excluded if they were experiencing severe mental health difficulties or other circumstances whereby participation in the study could exacerbate distress.

Recruitment took place online through social media, the Manchester Metropolitan University participation pool, relevant charities, and support groups between 31 March 2025 and 30 June 2025. The study was advertised by the first author through LinkedIn and Facebook support groups focused on endometriosis, vulvodynia, and bladder pain syndrome/interstitial cystitis. It was also disseminated through Bladder Health UK, the Menstrual Health Project, and the Pelvic Pain Support Network. Recruitment routes are summarised in Supplementary Table S1.

The survey link received 232 recorded entries. Of these, 85 were incomplete questionnaires that were not submitted to the end of the survey and were therefore deleted without being included in the analysis. The final analytic sample comprised 147 completed questionnaires. A priori power analysis indicated that 89 participants would provide 0.95 power to detect a medium effect (f^2^ = .15) in multiple regression with six predictors at *α* = .05.^36^ The achieved sample exceeded this threshold. Participant characteristics are presented in Table 1.

**Table 1.** Sample characteristics.

Variable	Category	n	%
Gender	Female	147	100.0
Ethnicity	White British	77	52.4
	White European	26	17.7
	Any other White background	13	8.8
	White Irish	11	7.5
	Any other ethnic group	7	4.8
	Filipino	2	1.4
	Indian	2	1.4
	Persian (Other ethnic group)	2	1.4
	White and Black Caribbean	2	1.4
	Any other mixed background	1	0.7
	Arab (Other ethnic group)	1	0.7
	Black African	1	0.7
	Chinese	1	0.7
	Pakistani	1	0.7

### Procedure

Potential participants accessed the study via a link or QR code in online materials. The participant information sheet described the study purpose, eligibility, confidentiality, and risks, and stated that responses were anonymous and could not be withdrawn after submission because no identifiers were collected. Participants provided electronic consent before accessing the questionnaire. After completion, a debrief page reiterated the voluntary nature of participation and provided support resources.

### Measures

**Pain management strategies:** Study-specific items were developed to assess pain management strategies and were not formally validated. Participants indicated all pain-management strategies they had used at any time. Response options included surgery, hormonal medication or therapies, analgesia/painkillers, non-hormonal medications/therapies, psychological therapies, and alternative therapies. Analgesia/painkillers referred to medicines used for pain relief, such as paracetamol, non-steroidal anti-inflammatory drugs, and codeine-based analgesics. Non-hormonal medications/therapies referred to non-hormonal approaches such as tricyclic antidepressants, muscle relaxants, and bladder treatments. Psychological therapies were presented with examples including cognitive behavioural therapy, multidisciplinary pain management programmes, and psychosexual counselling. This category therefore reflected participants’ self-reported recognition of psychological support and may not have captured psychologically informed elements embedded within multidisciplinary pain programmes, group interventions, or physiotherapy where these were not identified by participants as psychological therapy. The six strategy categories were recoded into binary indicators (0 = not used, 1 = used), allowing simultaneous modelling of overlapping strategy use. The survey did not capture whether strategies were current, their dose, duration, frequency, adherence, delivery format, or perceived helpfulness. Free-text entries involving “other” pain management strategies, and pain types, were coded to these categories using keyword rules. Where participants indicated use of alternative pain management strategies such as physiotherapy or acupuncture, these were retained for descriptive reporting.

**Quality of life (WHOQOL-BREF):** The WHOQOL-BREF^37^ was selected as it is a validated multidomain measure of quality of life that allows for the analysis of physical, psychological, social and environmental domains separately, which was considered appropriate for this heterogeneous sample. This contains 26 items scored 1 to 5 and produces four domain scores: Physical (7 items), Psychological (6 items), Social (3 items), and Environment (8 items).^37^ The Physical domain assesses areas such as pain, energy, sleep, mobility, daily activities and work capacity; the Psychological domain assesses positive and negative feelings, self-esteem, body image, thinking and concentration; the Social domain assesses personal relationships, social support and sexual activity; and the Environment domain assesses areas such as safety, home environment, financial resources, access to healthcare, leisure opportunities and transport. Items 3, 4, and 26 were reverse-scored prior to computing domain means. Domain means were then transformed to a 0 to 100 scale for interpretability, with higher scores indicating better QoL.^37^ The global facet item, Q1 (overall QoL), was analysed as an ordinal outcome. Subscale reliability scores were excellent, consistent with prior work^38^: Physical QoL α = .83, Psychological QoL α = .83, Social QoL α = .70, Environment QoL α = .85.

**Pain interference:** Pain interference was operationalised as the mean of WHOQOL-BREF items 3 and 4 in their raw, non-reversed direction. Item 3 asks, *“To what extent do you feel that physical pain prevents you from doing what you need to do?”* and item 4 asks, *“How much do you need any medical treatment to function in your daily life?”* Higher scores indicate greater interference.

### Statistical analysis

Analyses were conducted in jamovi (Version 2.3).^39^ The six binary strategy indicators were entered simultaneously as predictors in separate multiple linear regression models for each 0 to 100 WHOQOL-BREF domain and for pain interference. The global QoL item (Q1) was analysed with proportional-odds ordinal logistic regression using the same predictors. This approach estimated the unique association of each strategy while accounting for overlap among strategies.

For linear models, standard assumptions were examined. Residual normality and homoscedasticity were checked using Q-Q plots and residuals versus fitted values. Independence with Durbin-Watson statistics and multicollinearity with variance inflation factors were assessed. Influence diagnostics were reviewed to ensure that results were not driven by single cases. For the ordinal model, we assessed the proportional-odds assumption and interpreted results with caution where formal tests were limited in the software.

### Ethics

Ethical approval was granted by the Manchester Metropolitan University Health and Education Research Ethics and Governance Committee (approval ID: 75817) on 31 March 2025. Participation was anonymous. Participants could stop the questionnaire at any point before submission, and partial questionnaires were not included in the final dataset. Because the final dataset was anonymous, completed and submitted questionnaires could not be withdrawn after submission. The debrief information reiterated the voluntary nature of the study and provided support contacts. Data was exported from Qualtrics and stored on secure university storage with restricted access, in line with GDPR and institutional policy.

## Results

### Participants and sample characteristics

A total of 147 participants were recruited for this study. Participants’ ages ranged from 18 to 66 years (*n* = 147, *M* = 32.12, *SD* = 11.13). All respondents identified as female (100%, *n* = 147). The largest ethnic groups were White British (52.4%, *n* = 77), White European (17.7%, *n* = 26), and White Irish (7.5%, *n* = 11). Additional categories are shown in the table (See Table 1).

### Pain management strategies

Respondents reported a range of pain-management strategies (Table 2). The most frequently reported were analgesia/painkillers (78.2%, *n* = 115) and hormonal treatment/medication (74.8%, *n* = 110). Half reported non-hormonal medications/therapies (50.3%, *n* = 74) and about half reported surgery (49.7%, *n* = 73). Alternative medicine/treatment was reported by 42.9% (*n* = 63), and psychological therapies by 31.3% (*n* = 46).

**Table 2.** Pain management strategies reported (N = 147).

Strategy	n	%
Analgesia (painkillers)	115	78.2
Hormonal treatment/medication (e.g., the contraceptive pill, implant, hormone therapy, etc.)	110	74.8
Non-hormonal medications/therapies (e.g., tricyclic antidepressants, muscle relaxants, bladder treatments, etc.)	74	50.3
Surgery	73	49.7
Alternative medicine/treatment	63	42.9
Psychological therapies (e.g., CBT, psychosexual counselling, etc.)	46	31.3

*Note.* Percentages are of respondents and may exceed 100% because multiple responses were allowed.

### Clinical history

Symptoms typically began in late adolescence/early adulthood (age of onset: *n* = 144, *M* = 21.08, *SD* = 11.47). Among those with a recorded diagnosis age, diagnoses generally occurred in the mid-to-late 20s (age at diagnosis: *n* = 126, *M* = 28.22, *SD* = 10.55). For participants with a codable duration from point of first help-seeking to diagnosis, the average time to obtain a diagnosis was approximately 6-7 years (*n* = 104, *M* = 6.80, *SD* = 6.42). Among those still seeking a diagnosis, the mean time already spent seeking was just over five years (*n* = 35, *M* = 5.07, *SD* = 5.66). See Table 3.

**Table 3.** Clinical history: Age of onset, age at diagnosis, and time intervals.

Variable	N	M	SD	Range
Age of symptom onset (years)	144	21.08	11.47	6–59
Age at diagnosis (years)	126	28.22	10.55	14–62
Time to diagnosis (years)	104	6.80	6.42	0.02–36.00
Time seeking diagnosis still seeking (years)	35	5.07	5.66	0.00–20.00

*Note.* Non-duration responses were excluded, months/weeks converted to years.

#### Diagnoses (multi-response)

The most frequently reported condition was endometriosis (61.9%, *n* = 91), followed by vulvodynia (20.4%, *n* = 30), bladder pain syndrome/interstitial cystitis (10.2%, *n* = 15), and polycystic ovary syndrome (10.2%, *n* = 15). A subset reported Stage 4 endometriosis (4.1%, *n* = 6). Full counts, including less common diagnoses, and mean age of symptom onset for each condition reported, are provided in Table 4.

**Table 4.** Self-reported diagnoses among participants.

Diagnosis category	n	%	(age of symptom onset)
Endometriosis	91	61.9	17.6
Vulvodynia	30	20.4	23.6
Bladder pain syndrome/IC	15	10.2	29.1
PCOS	15	10.2	14.1
Suspected endometriosis	10	6.8	17.4
Pelvic floor dysfunction	8	5.4	21.9
CPP (unspecified)	6	4.1	21.0
Stage 4 endometriosis	6	4.1	18.3
Vaginismus	6	4.1	15.0
Other conditions	27	18.4	—

*Note.* Counts and percentages are based on multiple selections; categories with fewer than 2% frequency appear under “Other conditions.”

#### Symptom types

Common symptom types were sharp, stabbing pain (83.0%, *n* = 122), dull, aching pain (68.7%, *n* = 101), intermittent pain (62.6%, *n* = 92), stinging/burning/superficial pain (59.2%, *n* = 87), and constant/ongoing pain (52.4%, *n* = 77).

#### Average pain intensity (0-10)

Average pain intensity over the past three months was 6.84 out of 10 (*n* = 147, *M* = 6.84, *SD* = 2.24, median = 7.0, range = 0–10).

#### “Other” pain management subcategories

Among “Other” strategies (*n* = 49), the most frequent were herbal/natural remedies & supplements (24.5%, *n* = 12), acupuncture/Traditional Chinese Medicine (18.4%, *n* = 9), and pelvic-floor physiotherapy/rehab (18.4%, *n* = 9). Additional subtypes (e.g. physiotherapy, heat/cold, exercise/movement, diet, TENS, moisturisers/lubricants) are detailed in Table 5.

**Table 5.** Other pain management strategies (n = 49).

Subcategory	n	%
Herbal/Natural remedies & supplements	12	24.5
Acupuncture/Traditional Chinese Medicine	9	18.4
Pelvic floor physiotherapy/pelvic rehab	9	18.4
Physiotherapy/manual therapy (general)	4	8.2
Heat/cold modalities	4	8.2
Exercise/movement (incl. yoga, stretching)	3	6.1
Other	3	6.1
Diet/nutrition	2	4.1
TENS/electrical stimulation	2	4.1
Moisturisers/lubricants	1	2.0

### Descriptive statistics

WHOQOL-BREF domain scores were transformed to a 0-100 scale, with higher scores indicating better QoL. Domain scores were: Physical QoL (M = 39.6, SD = 18.9), Psychological QoL (M = 42.2, SD = 19.8), Social QoL (M = 44.6, SD = 24.0), and Environment QoL (M = 53.2, SD = 20.2). Pain interference was also transformed to a 0-100 scale, with higher scores indicating greater interference (M = 65.9, SD = 22.2). Within this sample, Physical and Psychological QoL were the lowest-scoring domains, while Environment QoL was the highest-scoring domain.

### Assumption checks

Across models, residuals were approximately normal (Shapiro-Wilk *p* > .05), residuals showed no pattern against fitted values (homoscedasticity), independence was adequate (Durbin-Watson = 1.77-2.14), and multicollinearity was low (VIF ≤ 1.17), and relationships between predictors and the outcome were approximately linear.

### Multiple regression models for QoL domains

As pain management strategies were recorded as having been used at any time, the following models examine associations between lifetime reported strategy use and current QoL and pain interference. Results should therefore be interpreted as cross-sectional associations rather than evidence that a strategy was currently being used or had a direct effect on current outcomes.

#### Physical QoL

The model was significant, explaining 13.6% of the variance (*F*(6, 140) = 3.68, *p* = .002, *R*^2^ = .136, adjusted *R*^2^ = .099). Surgery (*b* = -8.20, 95% CI [−14.14, −2.26], *p* = .007) and analgesia use (*b* = -8.16, 95% CI [−15.67, −0.65], *p* = .033) were associated with lower Physical QoL. Other strategies were not significant.

#### Psychological QoL

The model was significant, explaining 12% of the variance (*F*(6, 140) = 3.08, *p* = .007, *R*^2^ = .12, adjusted *R*^2^ = .079). Analgesia was associated with lower Psychological QoL (*b* = -9.34, 95% CI [-17.28, -1.40], *p* = .022), while psychological therapy was associated with higher Psychological QoL (*b* = 7.72, 95% CI [0.49, 14.95], *p* = .037). Other predictors were non-significant.

#### Social QoL

The overall model was not significant (*F*(6, 140) = 0.77, *p* = .596, *R* = .18, *R*^2^ = .032, adjusted *R*^2^ = -.010). No predictors reached *p* < .05.

#### Environment QoL

The model was significant (*F*(6, 140) = 2.36, *p* = .033, *R*^2^ = .092, adjusted *R*^2^ = .053). Psychological therapy was associated with higher Environment QoL (*b* = 8.72, 95% CI [1.23, 16.22], *p* = .023), whereas analgesia was associated with lower Environment QoL (*b* = -8.60, 95% CI [−16.83, −0.37], *p* = .041).

### Multiple regression model for pain interference

The overall model did not reach significance (*F*(6, 140) = 1.87, *p* = .090; *R* = .27, *R*^2^ = .074, adjusted *R*^2^ = .035). Non-hormonal medications/therapies was independently associated with higher pain interference (*b* = 9.41, 95% CI [1.81, 17.01], *p* = .016), no other predictors were significant.

### Ordinal logistic regression model for global QoL

The model was significant overall (χ^2^ (6)= 12.6, *p* = .049; McFadden *R*^2^ = .031; Nagelkerke *R*^2^ = .040), and met the proportional-odds assumption. No individual predictor reached *p* < .05. A visual summary of the adjusted associations observed across the regression models is presented in Figure 1.

**Figure 1.**
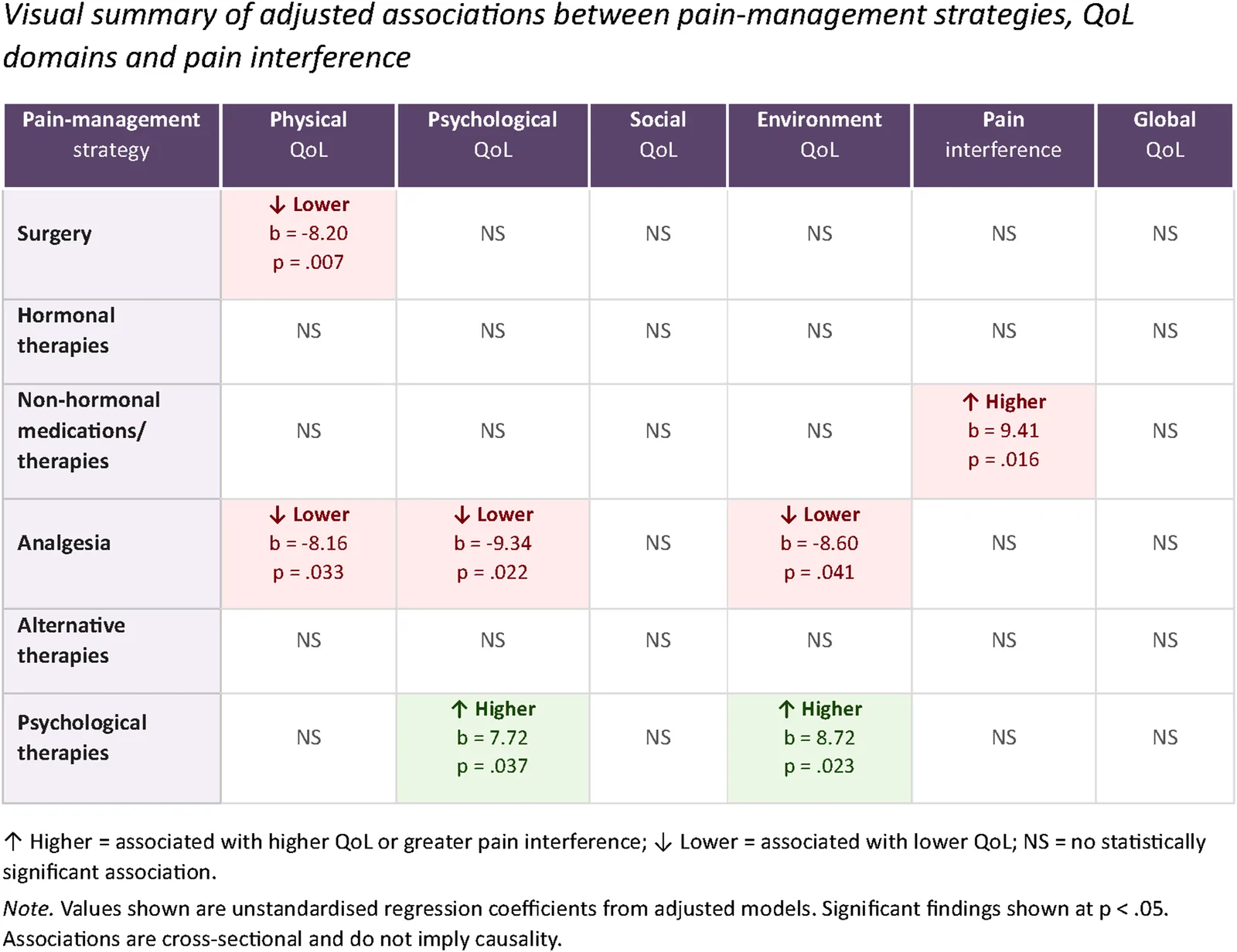
Visual summary of adjusted associations between pain-management strategies, QoL domains and pain interference.

## Discussion

This study examined the extent to which commonly used pain-management strategies are associated with pain interference and quality of life in women with chronic pelvic pain. Surgery was associated with poorer physical QoL. Reported use of analgesia was associated with poorer physical, psychological, and environmental QoL. Psychological therapies were associated with better psychological and environmental QoL. Use of non-hormonal medications/therapies was associated with greater pain interference. No strategy showed a consistent association with the social QoL domain. These findings fit a biopsychosocial understanding of pelvic pain, whereby use of biomedical treatments may reflect higher symptom severity, while psychological support was associated with higher psychological and environmental QoL.

### Surgery and physical quality of life

Women who reported having had surgery to manage their pain reported lower physical quality of life. This is unlikely to reflect surgery “causing” poorer outcomes and more likely reflects who is selected for surgery i.e. people with the most complex disease or poorer functioning are often the ones offered surgical intervention (confounding by indication), making it difficult to separate treatment effects from baseline severity.^40–42^ Indeed, studies highlight that improvements in quality of life vary depending on phenotype, central sensitisation, and comorbid pain mechanisms.^17,43^ Even when surgery is technically successful, neuropathic or centralised pain can limit gains,^44^ and many women need repeat operations within a few years.^16,43^ In chronic pain and endometriosis, this pattern is common and expected in cross-sectional research,^40–42^ and highlights why surgery may have been linked to lower physical QoL in this study (i.e. patients who need surgery often have more complex or long-standing pain, meaning that surgical treatment alone may not resolve the wider pain mechanisms that influence everyday functioning).

### Analgesia and lower quality of life

Analgesic use was consistently linked to poorer physical, psychological, and environmental quality of life. This may reflect symptom severity and treatment burden rather than medication having a damaging effect on quality of life by itself. However, side effects from commonly used medicines, including sedation, gastrointestinal problems, cognitive dulling, and nausea, can interfere with mood, mobility, social independence, and daily structure which may contribute to poorer physical, psychological and environmental QoL.^19,45–48^ CDC and NICE guidelines distinguish between pharmacological strategies more broadly and long-term opioid use specifically. Long-term opioid use for chronic pain has limited evidence of sustained benefit and carries risks including dependence, overdose, tolerance and adverse effects, whereas non-pharmacological approaches are recommended as important components of chronic pain care rather than as replacements for all pharmacological treatment.^11,22^ This finding may be further compounded by variable adherence rates, which can be influenced by side effects, beliefs about treatment, and treatment fatigue.^49,50^ In the present study, however, the analgesia category was broad and was not limited to opioids. The observed associations should therefore be interpreted as reflecting self-reported analgesic use overall, rather than evidence about any specific medicine class.

### Psychological therapy and quality of life

Psychological therapy was associated with better psychological and environmental QoL. However, this association should be interpreted cautiously, as psychological therapy was the least frequently reported strategy category and may have included varied forms of support. This is consistent with evidence for CBT, ACT, and broader biopsychosocial approaches in chronic pain, including for endometriosis and vulvodynia.^25–29^ These therapies target mood, worry, avoidance, sleep, pacing, and values-based action rather than pain intensity alone, with evidenced improvements mirroring the findings of the present study. Despite this evidence, psychological support is still underdelivered for pelvic pain in the UK. NCEPOD and Endometriosis UK both highlight challenges to accessing psychological therapies for people affected by endometriosis and pelvic pain, and a lack of routine holistic assessment.^8,9^ These findings suggest that earlier access to psychological support within CPP care pathways warrants further consideration and evaluation, while recognising that people with higher QoL may also be better able to access or engage with psychological therapy. Reverse causation could therefore partly explain the observed association.

### Non-hormonal medication/therapies and higher pain interference

Participants indicating use of non-hormonal medications/therapies reported higher pain interference. The EAU guideline notes that evidence for several non-hormonal pharmacological approaches in CPP is limited and variable, and many women continue to experience high interference even when pain intensity changes modestly.^23,51^ Side effects such as sedation or dizziness can affect daily functioning, contributing to interference even when pain intensity is partially managed. As such, this pattern may reflect a more severe clinical profile, and highlights the need to consider patient preferences and goals beyond pain intensity when considering pharmacological options to support pain management.

### Clinical and service implications

These findings suggest several areas for further clinical consideration within CPP care. The observed associations of both surgical intervention and analgesic use with poorer QoL suggest that commonly used biomedical strategies may not, in isolation, be sufficient to improve daily functioning and wellbeing and may reflect higher symptom severity or unmet clinical need. In particular, the associations between analgesia use and poorer physical, psychological, and environmental QoL highlight the importance of regularly reviewing pain management plans, monitoring broader impacts beyond pain intensity (e.g. functional goals such as sleep, mobility, and participation), and considering potential adverse or burdensome effects of long term medication use.

Early access to pelvic health physiotherapy, psychological input, and education aligns with national guidelines but remains inconsistent.^8–11^ The positive associations between psychological therapies and psychological and environmental QoL in the present study suggest that psychological support may be an important component to consider within routine CPP care, although longitudinal and intervention research is needed to clarify its role and timing. These findings align with a biopsychosocial model, highlighting the value of multidisciplinary approaches that address cognitive, emotional, and contextual factors alongside physical symptoms. Overall, these results are consistent with the value of holistic, personalised care pathways that balance symptom management with strategies aimed at improving broader QoL outcomes.

### Strengths and limitations

This is one of the first studies to examine multiple pain-management strategies simultaneously in women with CPP using validated multidomain QoL outcomes. By modelling overlapping strategy use, the study was able to estimate the unique associations of each strategy while reflecting the reality that many women use more than one approach to manage pelvic pain. A further strength is the focus on QoL and pain interference rather than pain intensity alone, which captures broader aspects of daily functioning that are highly relevant to CPP care. The heterogeneous sample also reflects the clinical reality of CPP, where women may have diagnosed, suspected or uncertain presentations. Internal consistency of the WHOQOL-BREF domains was good, and the pain interference composite performed well.

Several limitations should be considered when interpreting the findings. Causality cannot be inferred from the cross-sectional design, and confounding by indication is likely, as treatment use may reflect underlying symptom severity, treatment complexity or clinical need.^40–42^ Treatment strategies were also measured as having been used at any time, whereas QoL and pain interference reflected current functioning. The analyses therefore could not determine whether strategies were current, perceived as helpful, or temporally related to present outcomes. The binary operationalisation of strategies also meant that dose, duration, frequency, adherence, delivery format and treatment combinations could not be examined.

CPP status and diagnoses were self-reported and were not clinically verified, meaning diagnostic misclassification is possible within this heterogeneous sample. Residual confounding is also possible because the models did not adjust for clinical factors such as pain severity, symptom duration, diagnosis subtype, comorbidities or underlying pain mechanisms. The modest variance explained by the regression models suggests that pain-management strategy use accounts for only part of the variation in QoL and pain interference, alongside broader clinical, psychological, social and service-related factors.

Online recruitment through social media, patient-support communities, charitable organisations and a university participant pool may have overrepresented participants with more severe, long-standing or support-seeking experiences of CPP. Recruitment source was not recorded at the individual level, preventing calculation of route-specific response rates. The sample was also largely White, which limits generalisability to more ethnically diverse populations. Separately, the online recruitment strategy may limit generalisability to people who are less engaged with online support networks. Finally, psychological therapy was the least frequently reported strategy category (n = 46) and may have included varied forms of support, so associations involving this category should be interpreted cautiously.

The absence of consistent associations with social QoL suggests that social functioning may be influenced by factors beyond individual treatment strategies and warrants further attention. The WHOQOL-BREF social scale has only three items, overlooking key areas such as workplace disruption, sexual pain, and relational strain. These are key to CPP, particularly for conditions like endometriosis and vulvodynia.^52^ Disease-specific measures such as the EHP-30 capture these experiences more sensitively.^53,54^ Future work should include both generic and condition specific PROMs.

Alternative therapies in this study were coded as a single category, making interpretation difficult. Physiotherapy, pelvic floor treatments, and mindfulness-based approaches each have different evidence bases and combining them into one item within the present study may have reduced meaningful effects. For example, physiotherapy in particular shows potential, but high-quality trials are still limited.^55,56^

## Conclusion

This study examined six commonly used strategies for chronic pelvic pain-management. Four patterns stood out: (1) Surgery was linked to lower physical quality of life, (2) analgesia was linked to poorer physical, psychological, and environmental quality of life, (3) psychological therapy was associated with higher psychological and environmental quality of life, and (4) use of non-hormonal medications/therapies was associated with greater pain interference. No clear associations were found for the social quality of life domain.

These findings are consistent with a biopsychosocial view of chronic pelvic pain, with biomedical strategy use potentially reflecting greater symptom burden, treatment complexity, or confounding by indication, while psychological support was associated with higher psychological and environmental quality of life. The results point to areas that may warrant further clinical attention and research, including access to psychological therapy, regular review of medication use, more comprehensive peri-surgical support, and routine monitoring of quality of life and pain interference in women with CPP. Future longitudinal and intervention research should examine these strategies in more detail, including dose, duration, adherence, timing and combinations of treatments, to clarify whether integrated medical, surgical, physiotherapy and psychological approaches are associated with better QoL and pain-interference outcomes over time.

## Supplemental material

Supplemental material - Pain management strategies and their associations with quality of life and pain interference in women with chronic pelvic pain: A cross-sectional survey Supplemental material for Pain management strategies and their associations with quality of life and pain interference in women with chronic pelvic pain: A cross-sectional survey by Darya Ghodosi, Jasmine Heath Hearn in Women’s Health

Supplemental material - Pain management strategies and their associations with quality of life and pain interference in women with chronic pelvic pain: A cross-sectional survey Supplemental material for Pain management strategies and their associations with quality of life and pain interference in women with chronic pelvic pain: A cross-sectional survey by Darya Ghodosi, Jasmine Heath Hearn in Women’s Health

Supplemental material - Pain management strategies and their associations with quality of life and pain interference in women with chronic pelvic pain: A cross-sectional survey Supplemental material for Pain management strategies and their associations with quality of life and pain interference in women with chronic pelvic pain: A cross-sectional survey by Darya Ghodosi, Jasmine Heath Hearn in Women’s Health

## Data Availability

Due to the sensitive nature of the data, the datasets generated and/or analysed during the current study are not currently publicly available but may be available from the corresponding author on reasonable request, subject to ethical and institutional restrictions.*
