# Supplementary material for: Pain management strategies and their associations with quality of life and pain interference in women with chronic pelvic pain: A cross-sectional survey
Source: Womens Health (Lond). 2026 Oct 7;22:17455057261485419. doi: 10.1177/17455057261485419 (PMC13769806; doi:10.1177/17455057261485419)
Supplement: Supplementary file 1 [file sj-pdf-1-whe-10.1177_17455057261485419.pdf]

Supplementary Table S1. Recruitment routes and platforms/organisations

**Recruitment route**	**Platforms/organisations**
Social media and peer-support groups	LinkedIn, Facebook groups focused on endometriosis, vulvodynia and bladder pain syndrome/interstitial cystitis
Patient/community organisations	Bladder Health UK; Menstrual Health Project; Pelvic Pain Support Network
University recruitment	Manchester Metropolitan University participant pool

Note. Recruitment route was not recorded for individual participants, therefore, source-specific recruitment numbers and response rates could not be calculated.
