# Supplementary material for: Pain management strategies and their associations with quality of life and pain interference in women with chronic pelvic pain: A cross-sectional survey
Source: Womens Health (Lond). 2026 Oct 7;22:17455057261485419. doi: 10.1177/17455057261485419 (PMC13769806; doi:10.1177/17455057261485419)
Supplement: Supplementary file 2 [file sj-pdf-2-whe-10.1177_17455057261485419.pdf]

APPENDIX 8- THE WHOQOL-BREF

ABOUT YOU I.D. number

Before you begin we would like to ask you to answer a few general questions about yourself: by circling the correct answer or by filling in the space provided.

What is your **gender**? Male Female

What is your **date of birth**? ________ / ________ / ________
Day / Month / Year

What is the highest **education** you received?
None at all
Primary school
Secondary school
Tertiary

What is your **marital status**?
Single
Married
Living as married
Separated
Divorced
Widowed

Are you currently **ill**? Yes No

If something is wrong with your health what do you think it is?___________________________

Instructions

This assessment asks how you feel about your quality of life, health, or other areas of your life. **Please answer all the questions.** If you are unsure about which response to give to a question, **please choose the one** that appears most appropriate. This can often be your first response.

Please keep in mind your standards, hopes, pleasures and concerns. We ask that you think about your life **in the last two weeks.** For example, thinking about the last two weeks, a question might ask:

		Not at all	Not much	Moderately	A great deal	Completely
	Do you get the kind of support from others that you need?	1	2	3	4	5

You should circle the number that best fits how much support you got from others over the last two weeks. So you would circle the number 4 if you got a great deal of support from others as follows.

		Not at all	Not much	Moderately	A great deal	Completely
	Do you get the kind of support from others that you need?	1	2	3	4	5

You would circle number 1 if you did not get any of the support that you needed from others in the last two weeks. Please read each question, assess your feelings, and circle the number on the scale for each question that gives the best answer for you.

THE WHOQOL-BREF

		Very poor	Poor	Neither poor nor good	Good	Very good
1 (G1)	How would you rate your quality of life?	1	2	3	4	5

		Very dissatisfied	Dissatisfied	Neither satisfied nor dissatisfied	Satisfied	Very satisfied
2 (G4)	How satisfied are you with your health?	1	2	3	4	5

The following questions ask about **how much** you have experienced certain things in the last two weeks.

		Not at all	A little	A moderate amount	Very much	An extreme amount
3 (F1.4)	To what extent do you feel that (physical) pain prevents you from doing what you need to do?	1	2	3	4	5
4 (F11.3)	How much do you need any medical treatment to function in your daily life?	1	2	3	4	5
5 (F4.1)	How much do you enjoy life?	1	2	3	4	5
6 (F24.2)	To what extent do you feel your life to be meaningful?	1	2	3	4	5

		Not at all	A little	A moderate amount	Very much	Extremely
7 (F5.3)	How well are you able to concentrate?	1	2	3	4	5
8 (F16.1)	How safe do you feel in your daily life?	1	2	3	4	5
9 (F22.1)	How healthy is your physical environment?	1	2	3	4	5

The following questions ask about **how completely** you experience or were able to do certain things in the last two weeks.

		Not at all	A little	Moderately	Mostly	Completely
10 (F2.1)	Do you have enough energy for everyday life?	1	2	3	4	5
11 (F7.1)	Are you able to accept your bodily appearance?	1	2	3	4	5
12	Have you enough money to meet your	1	2	3	4	5

(F18.1)	needs?					
13 (F20.1)	How available to you is the information that you need in your day-to-day life?	1	2	3	4	5
14 (F21.1)	To what extent do you have the opportunity for leisure activities?	1	2	3	4	5

		Very poor	Poor	Neither poor nor good	Good	Very good
15 (F9.1)	How well are you able to get around?	1	2	3	4	5

The following questions ask you to say how **good or satisfied** you have felt about various aspects of your life over the last two weeks.

		Very dissatisfied	Dissatisfied	Neither satisfied nor dissatisfied	Satisfied	Very satisfied
16 (F3.3)	How satisfied are you with your sleep?	1	2	3	4	5
17 (F10.3)	How satisfied are you with your ability to perform your daily living activities?	1	2	3	4	5
18 (F12.4)	How satisfied are you with your capacity for work?	1	2	3	4	5
19 (F6.3)	How satisfied are you with yourself?	1	2	3	4	5
20 (F13.3)	How satisfied are you with your personal relationships?	1	2	3	4	5
21 (F15.3)	How satisfied are you with your sex life?	1	2	3	4	5
22 (F14.4)	How satisfied are you with the support you get from your friends?	1	2	3	4	5
23 (F17.3)	How satisfied are you with the conditions of your living place?	1	2	3	4	5
24 (F19.3)	How satisfied are you with your access to health services?	1	2	3	4	5
25 (F23.3)	How satisfied are you with your transport?	1	2	3	4	5

The following question refers to **how often** you have felt or experienced certain things in the last two weeks.

		Never	Seldom	Quite often	Very often	Always
26 (F8.1)	How often do you have negative feelings such as blue mood, despair, anxiety, depression?	1	2	3	4	5

Did someone help you to fill out this form?..

How long did it take to fill this form out?..
